# From bite to brain: Neuro‐immune interactions in food allergy

**DOI:** 10.1111/all.16366

**Published:** 2024-10-27

**Authors:** Vikki Houghton, Thomas Eiwegger, Esther Borges Florsheim, Rebecca C. Knibb, Sandrine Thuret, Alexandra F. Santos

**Affiliations:** ^1^ Peter Gorer Department of Immunobiology, School of Immunology and Microbial Sciences King's College London London UK; ^2^ Department of Basic and Clinical Neuroscience, Institute of Psychiatry, Psychology and Neuroscience King's College London London UK; ^3^ Department of Pediatric and Adolescent Medicine University Hospital St. Pölten St. Pölten Austria; ^4^ Translational Medicine Program, Research Institute The Hospital for Sick Children Toronto Canada; ^5^ Department of Immunology, Temerty Faculty of Medicine University of Toronto Toronto Canada; ^6^ Karl Landsteiner University of Health Sciences Krems Austria; ^7^ Center for Health Through Microbiomes Biodesign Institute Arizona State University Tempe Arizona USA; ^8^ School of Life Sciences Arizona State University Tempe Arizona USA; ^9^ Institute of Health and Neurodevelopment Aston University Birmingham UK; ^10^ Department of Women and Children's Health (Paediatric Allergy), School of Life Course Sciences, Faculty of Life Sciences and Medicine King's College London London UK; ^11^ Children's Allergy Service Guy's and St. Thomas' NHS Foundation Trust London UK

**Keywords:** anxiety, food allergy, immune mechanisms, mast cells, neuro‐immune interactions

## Abstract

Immunoglobulin E (IgE)‐mediated food allergies are reported to affect around 3.5% of children and 2.4% of adults, with symptoms varying in range and severity. While being the gold standard for diagnosis, oral food challenges are burdensome, and diagnostic tools based on specific IgE can be flawed. Furthering our understanding of the mechanisms behind food allergy onset, severity and persistence could help reveal immune profiles associated with the disease, to ultimately aid in diagnosis. Alterations to cytokine levels and immune cell ratios have been identified, though further research is needed to fully capture the heterogenous nature of food allergy. Moreover, the existence of such immune alterations also raises the question of potential wider systemic effects. For example, recent research has emphasised the existence and impact of neuro‐immune interactions and implicated behavioural and neurological changes associated with food allergy. This review will provide an overview of such food allergy‐driven neuro‐immune interactions, with the aim of emphasising the importance of furthering our understanding of the immune mechanisms underlying IgE‐mediated food allergy.

## 1 INTRODUCTION

Food allergies (FA) typically occur via two mediatory pathways; immunoglobulin E (IgE) mediated or non‐IgE‐mediated, that manifest as distinct clinical phenotypes.^1^ The most common form, IgE‐mediated FA, is considered to be rising in prevalence and severity, particularly in Western Europe.^2^, ^3^, ^4^ However, discrepancies in self‐reported, physician‐ and oral food challenge‐ (OFC) confirmed data lead to difficulties in quantifying the true incidences of FA.^5^, ^6^ Indeed, a recent systematic review^7^ revealed that, while physician‐ and OFC‐diagnosed prevalence of any FA were 6.6% and 0.8%, respectively, self‐reported figures were at 19.9%. Similarly, a population‐based survey of US adults estimated that 10.8% had at least one convincing FA, whereas self‐reported values were 19.0%.^8^ Differences between clinically‐confirmed and self‐diagnosed food allergy rates have been attributed to experiential factors, such as psychosomatic reactions or coincidental pairing of food consumption with symptoms, but also due to misdiagnosis given the scarcity of allergy services.^9^ These complications, alongside the differences in physician and OFC‐confirmed rates of FA, attest to how differences in disease manifestation and the associated challenges in diagnosis can obscure true rates of FA.

While OFCs are considered the gold standard method for FA diagnosis, they are also time‐consuming, costly and involve potential risk to the patient through the possibility of allergic reactions.^10^ Thus, FA diagnosis is often based on the combination of skin prick test (SPT) results and serum levels of allergen specific‐IgE (sIgE), along with patient history. Yet, sIgE levels and SPT results rely on IgE sensitisation, which does not necessarily correlate with clinical manifestations of FA.^11^, ^12^, ^13^, ^14^ SPT and sIgE diagnostic tests are reported to have low to moderate specificity and positive predictive value when compared to OFC diagnoses, and have been attributed to overdiagnoses.^12^, ^15^ Moreover, sIgE does not appear to be capable of predicting FA persistence or transience,^16^ and may only be minimally useful for predicting the severity of allergy and consequent reactions.^17^, ^18^, ^19^, ^20^ Consequently, identifying a greater range of FA‐associated markers would not only benefit diagnosis, but also aid patients in managing their allergy through greater understanding of its manifestation.

Furthermore, clarifying the mechanisms and markers associated with FA will help to uncover the wider, systemic effects of the disease. Numerous studies have described an association between FA and the nervous system, revealing bidirectional communication between mast cells and neurons, particularly within the enteric nervous system (ENS).^21^, ^22^ Such interactions of the immune and nervous system in FA appear to play a role in influencing symptoms experienced following allergen exposure,^23^ as well as further downstream behavioural effects.^24^, ^25^ Nonetheless, discrepancies across the literature regarding the occurrence and impact of neuro‐immune interactions present a gap in our current knowledge.

To this end, this review will evaluate the current research on neuro‐immune interactions in FA, in context of our current understanding of FA immune mechanisms. By exploring the wider effects of FA, we aim to highlight the importance of understanding such processes to minimise the systemic effects and reduce the disease burden.

## 2 NEURO‐IMMUNE INTERACTIONS ASSOCIATED WITH FOOD ALLERGY AND FOOD ALLERGIC REACTIONS

**FIGURE 1:**
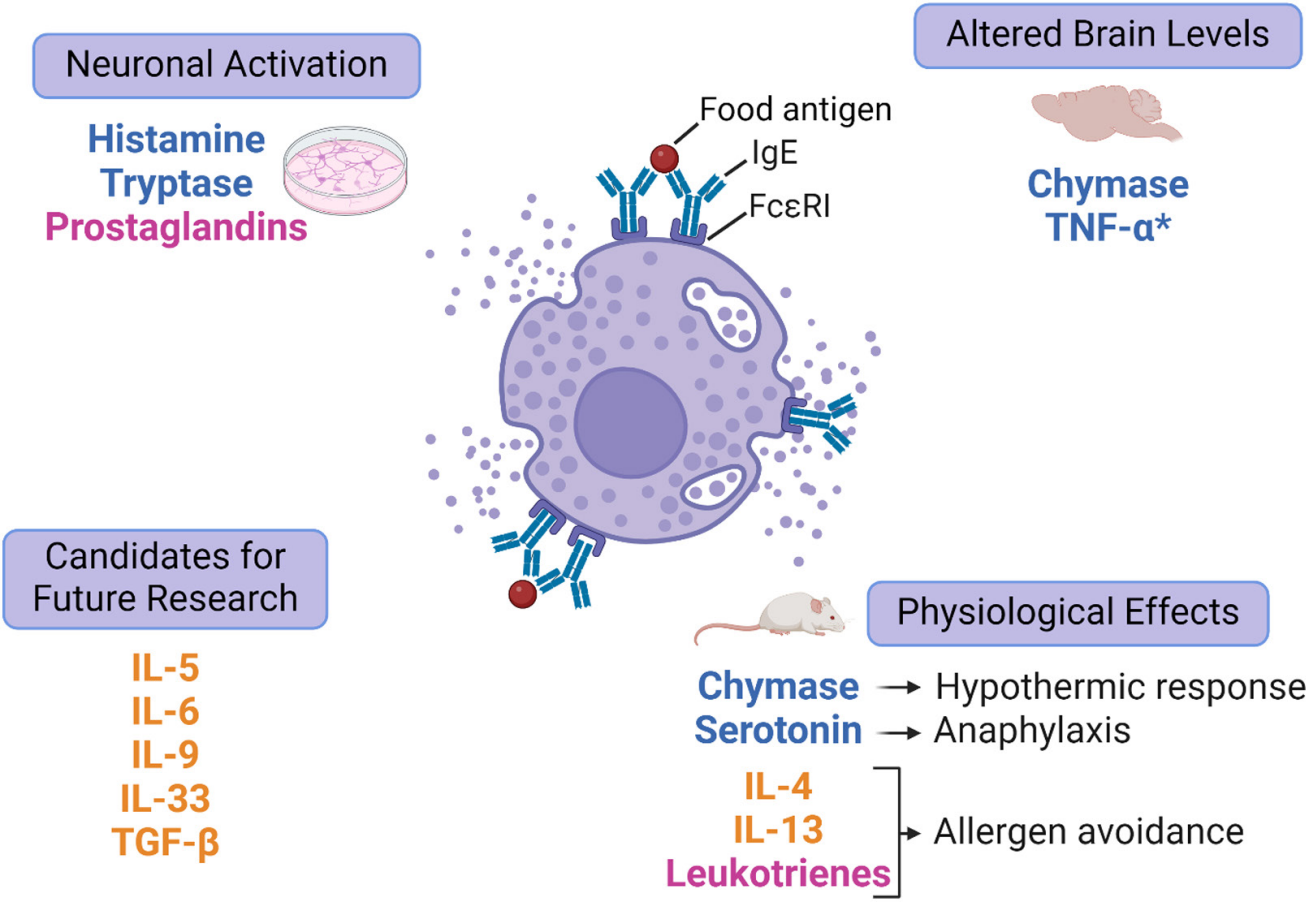
Mast cell mediators and associated neuronal interactions. A visual summary of the mast cell mediators outlined in this review that have been shown to directly activate neurons and/or produce a physiological effect. Candidates for future research have also been identified from common mast cell mediators where there is no evidence on the neuro‐immune interactions in the context of FA. Text colour indicates the stage at which the mediator is released upon degranulation: Blue represents preformed mediators, released rapidly upon degranulation; pink represents the release of rapidly synthesised lipid mediators; orange represents cytokines that are synthesised upon degranulation and subsequently released. *TNF‐α has been shown to be released immediately at degranulation, in a manner distinguishable to the other cytokines listed. FcεRI, Fc epsilon receptor 1; IgE, Immunoglobulin E; IL, Interleukin; TGF, Transforming growth factor; TNF, Tumour necrosis factor.

IgE sensitisation and the onset of FA are associated with a switch to a type‐2 inflammatory response, initiated through the epithelial release of pro‐inflammatory factors, including IL‐25, IL‐33 and TSLP.^26^ These mediators subsequently activate CD103+ dendritic cells^26^ (DCs) and cause them to release OX40L, which provokes naïve T cells to differentiate into Th2 cells.^27^ Th2 cells release further pro‐inflammatory factors, including IL‐4, that induce B cell class switching to produce IgE.^28^ IgE then binds to the high affinity Fc epsilon receptor 1 (FcεRI) on mast cells (MC) and basophils. Consequent exposure of these effectors cells to food antigen leads to cross‐linking of the IgE‐FcεRI complex, which, in some individuals, results in MC and basophil degranulation^29^ (Figure 1). This involves the rapid release of preformed mediators, including histamine, TNF‐α, tryptase and chymase, followed by the synthesis and release of prostaglandins and leukotrienes, and finally by cytokine production and release.^30^, ^31^

**FIGURE 2:**
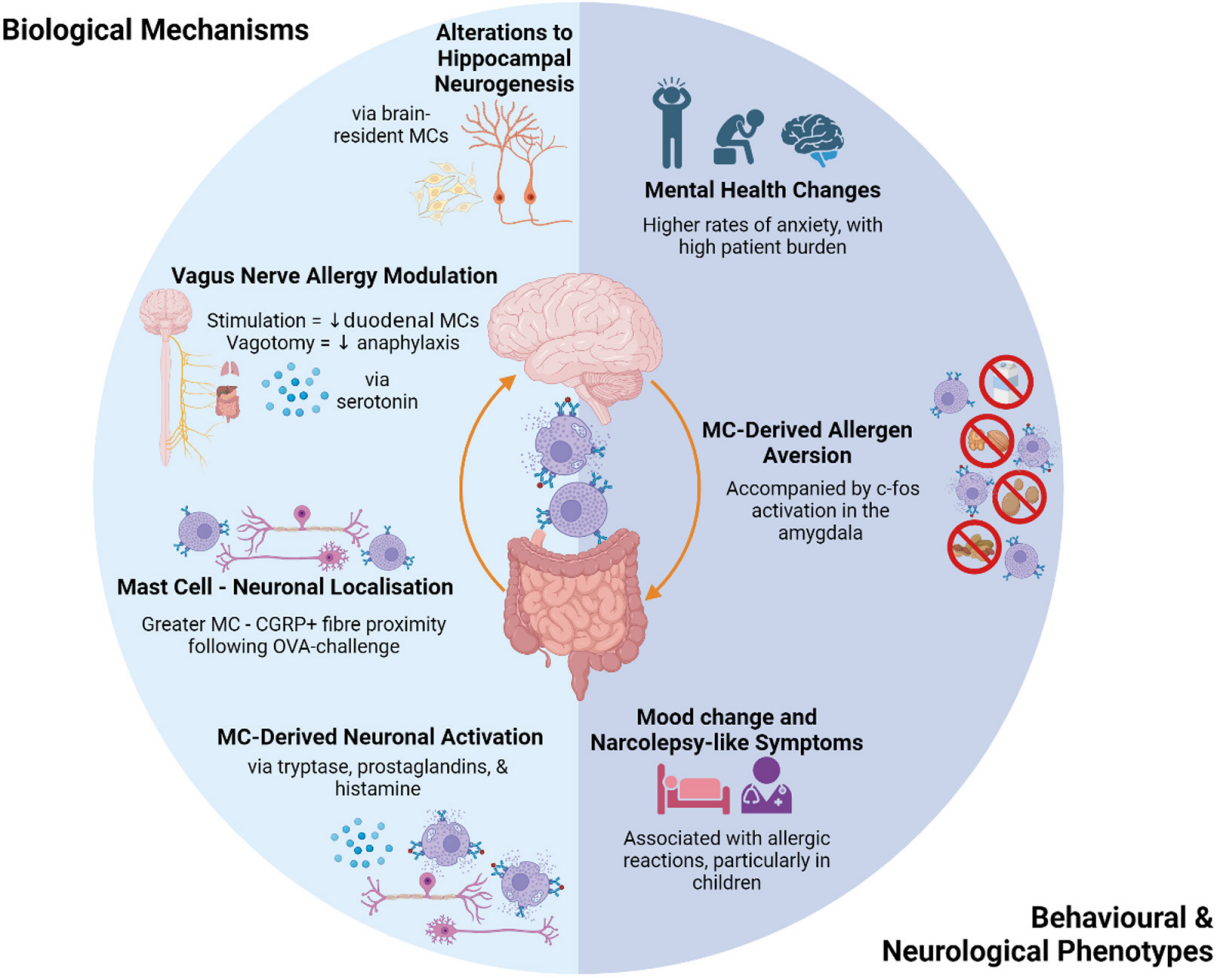
Summary of the neuro‐immune interactions associated with food allergy, split by biological mechanisms and behavioural & neurological phenotypes. All interactions outlined in this figure are described in greater detail in Section 2 of this review. All phenotypes are described as alterations in food allergy as compared to non‐allergic controls. CGRP, Calcitonin gene‐related peptide; CSF, Cerebrospinal fluid; MC, Mast cells.

This switch to a type‐2 inflammatory response, and the plethora of factors released by MCs during allergic reactions have generated interest in the neuro‐immune interactions associated with FA (Figure 2 and Box 1). These interactions are evidenced by the various neurological symptoms that can be experienced during allergic reactions, such as itchiness, sneezing, coughing and bronchoconstriction,^32^ and the wider literature has described neuronal interactions with eosinophils, type 2 innate lymphoid cells (ILC2s), and DCs. Eosinophils localise around neurons in the lung and nasal cavity following allergen challenge,^33^, ^34^ and in human atopic dermatitis skin.^35^ ILC2s express the receptor for calcitonin gene‐related peptide (CGRP),^36^, ^37^, ^38^ a neuropeptide involved in immune function,^39^ while the neuropeptide Neuromedin U activates ILC2s in vitro and amplifies allergic lung inflammation in vivo (when accompanied by IL‐25).^40^ Notably, TRPV1^+^ neurons can be directly activated by allergen,^41^, ^42^, ^43^ with Perner et al.^42^ reporting that direct neuronal activation by allergens was instrumental in triggering DC migration and, the consequent differentiation of Th2 cells.

BOX 1 **Examples of mast cell‐neuronal interactions**

1. Mast cells colocalise with neurons in the gut, with increased occurrence observed in allergic models.
2. Vagus nerve stimulation reduces the number and degranulation of MCs in food allergic rodent duodenal tissue.
3. Vagotomy prevents anaphylaxis in IgE‐sensitised rodents via serotonin signalling.
4. Tryptase stimulates Ca^2^^+^ signalling and results in the release of CGRP and substance.
5. Ex vivo human models demonstrate neuronal activation following exposure to supernatant from activated mast cells.
6. Mast cell depletion prevents the emergence of allergen avoidance behaviours in sensitised rodents.

To date, however, the focus of neuro‐immune interactions in allergy has been largely on asthma and dermatitis, with little on FA specifically. Yet, the fact that FA reactions often involve the gut, where the ENS is situated, indicates the possibility for such bidirectional interactions to occur. Indeed, mast cells are known as being in close proximity with neurons in the intestinal mucosa,^44^ as well as having the ability to bidirectionally communicate with them.^22^, ^45^ This effect appears to be exacerbated in FA, as supported by the reported increase of mast cell‐neuronal localisations in the gut of ovalbumin‐ (OVA) sensitised rodents compared to vehicle‐sensitised controls.^21^ Focusing on MC proximity to CGRP‐positive nerve fibres, Lee et al.^46^ indicated that the area of these fibres are positively correlated with the number of OVA challenges in FA mice. Interestingly, treating FA mice with a CGRP receptor antagonist reduced the number of MCs in the proximal colon and also attenuated allergy‐associated symptoms.^47^ While this may suggest an active role of MC‐neuronal proximity in FA, greater research is required before any such conclusion can be asserted.

The vagus nerve has been implicated as having a functional role in FA symptom severity via MC communication. Stimulation of the vagus nerve significantly reduced MC numbers in duodenal tissue and reduced mRNA expression of IL‐4, IL‐5, IL‐6, IL‐13 and TSLP.^23^ Notably, there was an accompanying reduction in plasma levels of mMCP‐1, a marker of MC degranulation, suggesting that the reduction in challenge‐associated inflammation was driven by vagal nerve‐mediated MC inhibition. Demonstrating the bidirectional nature of this communication, vagotomy was shown to prevent anaphylaxis in passive‐IgE‐sensitised mice, despite no differences in histamine levels.^45^ Of note, serotonin, another key MC‐derived mediator with well‐established neuronal actions, was identified as the key mediator driving anaphylaxis in this model, and its blockade led to similar results as vagotomy.

Evidence for MC‐neuronal communication is not limited to the vagus nerve, though most of it centres on a unidirectional perspective of neuronal modulation by MCs. In cultured rat dorsal root ganglia (DRG) neurons, the mast cell mediator tryptase was shown to activate protease‐activated receptor‐2, that led to the Ca^2+^‐dependent release of CGRP and substance P.^48^ Interestingly, the authors went on to demonstrate that the PAR2‐dependent release of such factors from spinal afferent neurons induced oedema in the rat paw via mast cell degranulation. Another study demonstrated ex vivo cholinergic neuronal activation following β‐Lactoglobulin (βLG) incubation in submucosa/mucosa tissue from βLG‐sensitised rodents, but not from non‐sensitised, control animals.^49^ Blockading prostaglandin synthesis or histamine receptors H_1_ and H_2_, independently or altogether, significantly reduced the short‐circuit current response following βLG incubation, implicating a role of these MC mediators in the neuronal antigen response. ex vivo experiments have also provided preliminary evidence of neuronal activation by MCs in humans, with one study reporting an excitatory response (i.e. action potential) in 31% of enteric neurons following exposure to supernatant from activated human MCs.^50^ While these ex vivo studies do not offer insight into the physiological consequences of such MC‐derived neuronal activation, they provide proof‐of‐concept of such interactions and warrant further in vivo research to understand the downstream effects.

One such study that identifies a physiological function of MC‐neuronal interactions comes from Bao et al.,^51^ who revealed a role of MC‐derived chymase as a neuronal modulator in the context of the hypothermic response associated with IgE‐mediated anaphylaxis in mice. Specifically, both chymase‐deficient Mcpt4^−/−^ mice and wild‐type mice treated with a chymase antagonist demonstrated an attenuated drop in core body temperature following OVA challenge. Similarly, blocking PAR1, a chymase receptor, expression in TRPV1^+^ neurons prevented their activation. While focused primarily on MC to neuronal unidirectional interactions, the authors also identified that, while chymase exposure did not directly lead to neuropeptide release, it did prime DRG neurons to release CGRP following histamine exposure, demonstrating the possibility of bidirectional interactions in the context of food allergy.

Of particular note from Bao et al., however, is the observation that wild‐type mice who experienced IgE‐mediated anaphylaxis displayed greater c‐*fos* activation in the brain parabrachial nucleus, a region associated with thermoregulatory signalling, compared to both MC‐deficient mice challenged with OVA and vehicle‐challenged controls. Whereas most research on neuro‐immune interactions in FA to date have typically focused on the peripheral nervous system, these results indicate that the central nervous system, including the brain itself, may also undergo FA‐associated alterations. Indeed, increased levels of IgE, IgG1 and IgG2a, as well as chymase and TNF‐α levels, both mast cell mediators, have been observed not only in the serum, but also in the brain of OVA‐sensitised mice, specifically in the cerebral cortex^52^ and the hippocampus.^53^ Intriguingly, the former study reported both elevated numbers of microglia and the percentage of those activated within the cerebral cortex and hippocampus of FA mice, with TNF‐α showing limited co‐localisation with Iba1^+^ cells, suggesting an alternative source of this cytokine.^52^ However, the exact impact of food allergies on the central nervous system necessitates a deeper understanding of blood–brain barrier permeability during allergen challenges, an area yet to be fully explored.

**FIGURE 3:**
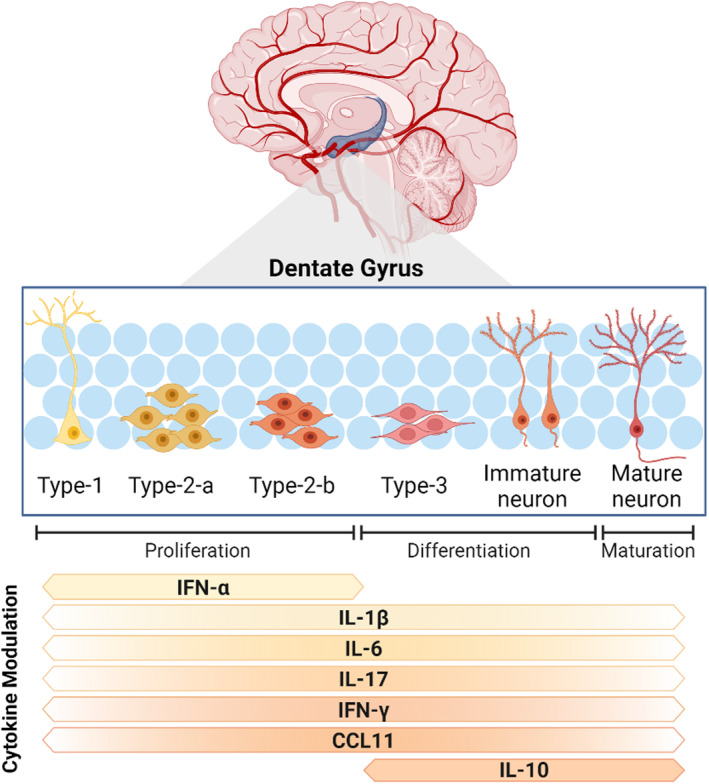
Adult Hippocampal Neurogenesis (AHN). Occurring along a highly vascularised niche, AHN proliferation, differentiation and maturation occurs along six stages, each characterised by a unique cell type. Proliferation, cell fate and survival can all be influenced by the systemic milieu. Cytokines have widely been described as playing a modulatory role on AHN across the cell life cycle, as indicated by the orange bars at the bottom of the image. CCL, C‐C motif chemokine; IFN, Interferon; IL, Interleukin.

The hippocampus may be prone to FA‐related alterations, as MCs appear to contribute to normal hippocampal function. Up to 25% of total brain mast cells reportedly reside in the hippocampus, with their degranulation providing a significant source of serotonin in the region.^54^ In particular, MCs may regulate adult hippocampal neurogenesis (AHN), the continuation of newborn neurons postnatally and throughout life^55^, ^56^ (Figure 3), under homeostatic conditions. Using the *W* ^*sh*^ */W* ^*sh*^ *and W* ^*sh*^/+ MC knockout (KO) lines, one study revealed reduced doublecortin (DCX, a neuroblast marker) immunoreactivity in MC‐deficient mice compared to those with brain‐resident MCs only.^54^ However, another study using an alternative MC KO model found no changes to AHN, despite observing decreased proliferation and increased neuronal‐lineage differentiation following in vitro co‐culture of hippocampal‐derived neural precursors and peritoneal MCs.^57^ Nonetheless, little research has investigated the effects of MC activation and degranulation on AHN, which would convey more relevance to a FA environment. One study, using a timothy grass pollen allergy model, demonstrated increased DCX and NeuN‐ (a marker of mature neurons) positive cells in the hippocampal dentate gyrus, compared to non‐allergic controls.^58^ While the exact functions of allergy‐induced AHN remain unclear, in non‐allergy settings, this process has been widely associated with specific forms of memory.^59^, ^60^, ^61^ Similar alterations have been alluded to in food allergy, with the literature indicating increased repetitive behaviours in cow's milk‐allergic mice, with allergic mice being shown to spend more time on self‐grooming and to be less likely to alternate arm in the T‐maze than control mice.^62^, ^63^ Similarly, a positive correlation between allergic tendency (not FA‐specific) and spatial ability has been reported in human adults.^64^ Nevertheless, further research will be instrumental in clarifying the role of AHN in food allergy.

Besides memory alterations, evidence links FA with various disease‐associated behavioural changes, further stressing the need to understand its broader impacts. Neurological symptoms often accompany food allergic reactions, with one study revealing such symptoms in 40% of patients (with a history of an allergic reaction).^65^ Specifically, 40% described feelings of unease, irritability, or anxiety, while 20% of children under six were reported as having a sudden behavioural change associated with their allergic reaction. Recently, Kalb et al.^66^ systematically investigated narcolepsy‐like sleepiness as a symptom in FA, reporting its manifestation in 12.5% of children during OFCs, and its significant association with eczema and allergens such as hazelnut and other tree nuts. This supports that narcolepsy‐like sleepiness is common in food allergies and is significantly associated with mild to severe allergic reactions, echoing findings from a previously published case study.^67^ Currently an underexplored aspect of food allergic reactions, the underlying biological mechanisms of these symptoms remain poorly understood.

Beyond allergic reactions, studies using allergen‐sensitised rodents describe behavioural alterations, specifically in the context of allergen‐avoidance,^68^, ^69^, ^70^, ^71^ as measured using the two‐bottle preference test. In particular, both the Plum et al.^68^ and Florsheim et al^69^ studies describe the rapid emergence of OVA avoidance in OVA‐sensitised mice, in contrast to the observed preference for OVA by their control counterparts. Mechanistically, this phenotype appears to be not only IgE‐dependent, as demonstrated by both studies using IgE KO mice, but also MC‐dependent, where MC‐KO transgenic mice^68^ and diphtheria‐driven MC‐depleted mice^69^ did not demonstrate allergen avoidance. Specifically, both studies highlight the role of leukotriene synthesis in the onset of this behavioural phenotype, as its blockade resulted in reduced allergen avoidance. Yet the mechanisms by which leukotrienes induce behavioural change remain unclear. Specifically, Plum et al. excluded the involvement of TPRV^+^ sensory neurons, and both studies reported that vagotomy had no impact on allergen avoidance behaviour, suggesting the involvement of other pathways downstream of mast cell activation. Florsheim et al. revealed the involvement of growth and differentiation factor 15 (GDF15), a TGF‐β cytokine family member, by demonstrating that MC‐depleted mice displayed rescued allergen avoidance behaviour following treatment with recombinant GDF15. Notably, the observed increase of *Gdf15* mRNA in the colon of OVA‐challenged BALB/c mice was shown to be derived from EPCAM^+^ epithelial cells in an IgE‐ and MC‐dependent manner, revealing the potential for wider circuitry involvement in FA neuro‐immune interactions. Furthermore, the Florsheim et al. and Lemos et al.^70^ studies also provide evidence of alterations beyond the gut with their findings that allergen‐challenged mice displayed increased c‐*fos* activation in the brain, particularly in the central amygdala, an area associated with avoidance behaviour in asthma^24^ and stress.^72^, ^73^ Both studies suggested that these changes exist as a defence mechanism to reduce harmful effects of allergen consumption, thereby indicating an evolutionary function of neuro‐immune interactions in food allergy.

Nevertheless, not all behavioural changes associated with FA are necessarily beneficial for patients, particularly when focusing on mental health. Patients with FA are largely reported to have worse health‐related quality of life^74^, ^75^, ^76^, ^77^ and higher risk and levels of anxiety.^78^, ^79^, ^80^, ^81^, ^82^ It has been suggested that increased anxiety in food allergic patients may be associated with increased vigilance,^83^, ^84^ thus serving as an adaptive coping strategy to aid with behaviours such as label reading and safe food choices.^79^, ^85^ However, one study found no association between adherence to medical management or “risk taking behaviour” for nut allergic children or their caregivers,^86^ and others have indicated that such safe, FA‐associated behaviours negatively impact quality of life.^87^, ^88^ Considering that peanut allergy patients and caregivers reported their mental and psychological health as being more greatly affected than their physical health,^77^ understanding the underlying biological mechanisms may be critical for future therapeutical targets. This is all the more emphasised with the observation that rodents with induced sensitisation go on to exhibit anxiety‐like behaviours.^25^, ^89^, ^90^ For example, compared to controls, OVA‐challenged, allergic mice were shown to spend significantly less time exploring in the elevated plus maze (EPM),^89^ a behaviour indicative of greater anxiety. They also displayed increased c‐*fos* expression in the amygdala and the paraventricular hypothalamic nucleus (PVN), a region associated with anxiety behaviours,^91^ via the involvement unmyelinated C‐fibres.^92^ Yet, further research is required to understand the extent of the neuro‐immune circuitry involved in the onset of FA‐specific anxiety.

Further research has uncovered effects beyond FA diagnosis, with one study demonstrating significantly elevated levels of phosphorylated tau in the hippocampus and parietal cortex of OVA‐allergic mice compared to non‐allergic controls.^53^ Given that hyperphosphorylated tau aggregations are a hallmark of Alzheimer's Disease (as reviewed by Noble et al.^93^), these results align with those from a longitudinal study of 22,188 twins that uncovered a 16% increase in risk of Alzheimer's or any dementia in individuals with a history of atopic disease.^94^ Contrarily, another study revealed a potential protective effect of FA on Alzheimer's risk, as those with a diagnosed or self‐reported allergy (including, but not limited to, FA) exhibited higher cognitive scores and reduced cerebrospinal fluid levels of amyloid‐β_42_,^95^ a controversial marker of amyloidosis.^96^ Despite uncovering wider behavioural and neurological effects of FA, the scarcity of research and existing discrepancies emphasise the need for further investigation into this area.

## 3 BEYOND sIgE: IMMUNE PROFILES OF FOOD ALLERGY AND FOOD ALLERGY REACTIONS IN HUMANS

**FIGURE 4:**
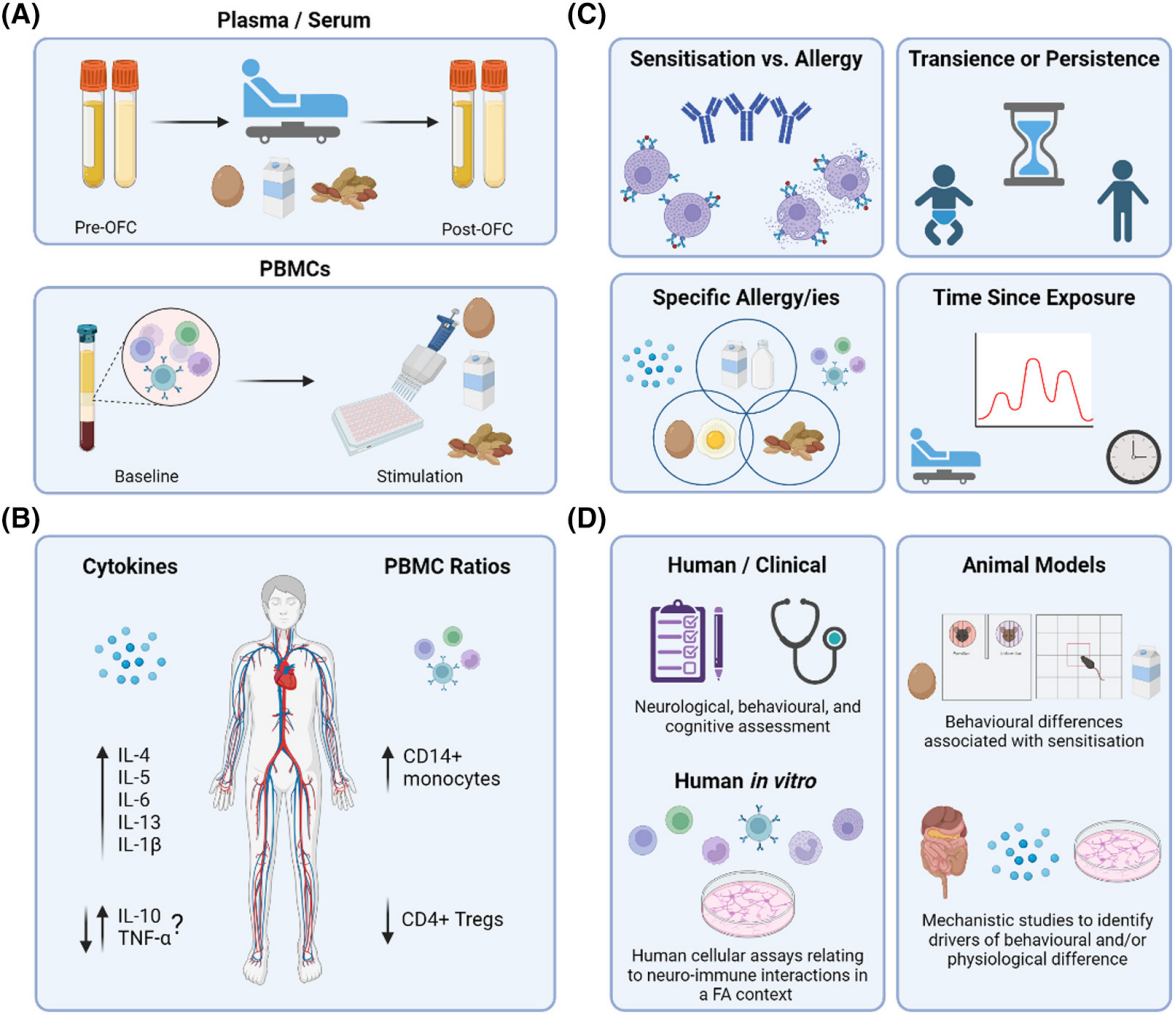
Exploring cytokine profiles associated with food allergy. (A) Common methods of measuring cytokine profiles in allergic and control individuals. Plasma and serum are analysed for analytes of interest, often with samples being collected before and after a double‐blinded, placebo‐controlled food challenge. Alternatively, peripheral blood mononuclear cells (PBMCs) can be collected, and expression levels can be measured before and/or after stimulation, often with allergen or lipopolysaccharide (LPS). (B) Summary of cytokine and PBMC ratios associated with food allergy. IL‐10 and TNF‐α indicate current disparity across the literature. (C) Common factors that modify cytokine profiles and/or PBMC ratios in food allergy, indicating areas that require greater research. (D) Research methods and avenues to ensure a clear understanding of neuro‐immune interactions in food allergy and the subsequent physiological and behavioural outcomes. FA, Food allergy; IL, Interleukin; OFC, Oral food challenge; PBMCs, Peripheral blood mononuclear cells.

To elucidate the broader effects of FA on the wider system, it will be important to first strengthen our understanding of FA mechanisms and to identify immune patterns, or profiles, associated with FA patients (Figure 4). Here, we discuss emerging FA profiles, to evaluate them in context of existing neuro‐immune interactions but also to highlight current gaps in our knowledge and potential avenues for future research.

### 3.1 Cytokines

With an interest in identifying FA‐specific immune landscapes and profiles, many studies have focused on cytokine levels (Table 1). The T helper 2 response associated with FA involves increased expression of the Th2 cytokines IL‐4, IL‐5 and IL‐13. Levels of these cytokines in FA individuals have been measured through various methods, including direct measurements in plasma or serum and ex vivo measurements from stimulated patient peripheral blood mononuclear cells (PBMCs). For instance, IL‐4 and IL‐13 levels were elevated in plasma from sensitised‐allergic (SA) patients, compared with non‐sensitised, non‐allergic (NSNA) controls.^97^ Such differences were observed between the sensitised, non‐allergic (SN) group compared with controls, indicating that these cytokines may primarily indicate IgE sensitisation, rather than allergy. Equally, IL‐5 was increased in plasma from patients with a suspected FA, regardless of OFC outcome, compared to non‐allergic healthy controls.^105^ These findings suggest that Th2 cytokines offer no greater insight into potential allergy beyond current sIgE measurements.

**TABLE 1:** A summary of reported cytokine alterations associated with food allergy.

Direction altered in FA^a^	Mode of measurement	Allergy	References
IL‐4			
↑	Plasma	Single egg/peanut^b^	^97^
	Serum	Unspecified^c^	^98^
	C/PBMC expression	Peanut	^99^
		Unspecified^c^	100, 101
IL‐13			
↑	Plasma	Single egg/peanut^b^	^97^
	PBMC expression	Peanut	^99^
		Milk	102, 103 ^d^
		Unspecified^c^	100, 104
IL‐5			
↑	Plasma	Peanut	^105^
	Serum	Unspecified^c^	^98^
	PBMC expression	Milk	^106^ ^d^
IL‐10			
↓	Plasma	Single egg/peanut^b^	^97^
	C/PBMC expression	Egg	^107^ ^d^
		Milk	^102^
		Unspecified^c^	^104^
↑	Plasma	Peanut	^105^
	PBMC expression	Peanut	99, 108
		Egg	^103^
		Milk	^108^ ^d^
		Unspecified^c^	^100^
IL‐1β			
↑	Serum	Unspecified^c^	^109^
	C/PBMC expression	Peanut	^108^
		Egg	^103^
		Unspecified^c^	100, 101
IL‐6			
↓	Plasma	Single egg/peanut^b^	^97^
↑	C/PBMC expression	Peanut	^108^
		Egg	^103^
		Unspecified^c^	100, 101
TNF‐α			
↓	PBMC expression	Egg	^107^ ^d^
↑	PBMC expression	Peanut	^108^
		Egg	^103^
		Unspecified^c^	100, 101

^a^ As compared to non‐allergic controls.

^b^ Single‐egg or ‐peanut participants were grouped together.

^c^ Signals studies where patients are grouped as ‘food allergic’ regardless of specific allergens.

^d^ Participants may have had multiple food allergies.

Nevertheless, mRNA analysis from unstimulated PBMCs collected at baseline—thus reflecting resting state rather than an allergic reaction—indicate that this may not always hold true. IL‐13 levels were significantly higher in children with a suspected FA (both SA and SN) compared to healthy controls, with greater elevation seen in those with a consequent positive double‐blinded, placebo‐controlled, food challenge (DBPCFC), compared to those with a negative challenge.^100^ Furthermore, mRNA expression of both IL‐4 and IL‐13 increased tenfold in individuals who experienced anaphylaxis during the challenge. While mRNA extraction would not be a cost‐effective diagnostic tool, these studies highlight differences in inflammatory status between sensitised and non‐sensitised patients, and potentially between SA and SN patients, raising questions about the downstream effects of these inflammatory changes. Notably, Florsheim et al.^69^ attributed IL‐4 and/or IL‐13 with the emergence of allergen avoidance, using IL‐4RA KO, OVA‐sensitised mice, indicating potential effects of these cytokines on behaviour. Moreover, along with their findings of increased neurogenesis, Klein et al.^58^ also reported increased serum levels of IL‐5 and IL‐13 in allergic mice, highlighting a potential the relationship between these observed changes and thus emphasising the need for greater research to address this knowledge gap.

Evidence for altered cytokines associated with FA extend beyond the Th2 response. One of common interest is IL‐10, a cytokine involved in the regulation of both gut homeostasis^110^ and AHN.^111^, ^112^ Yet, the results are discordant, with some studies describing decreased levels,^97^, ^102^, ^103^, ^104^, ^107^, ^113^ while others have reported increased levels of IL‐10^100^, ^105^, ^106^, ^108^ in FA, in a manner unrelated to the mode of measurement. There does, however, appear to be an effect of the specific allergen of interest, with egg allergic participants having reduced plasma levels compared with peanut allergics.^97^ Of particular note, two papers from the same team with similar methodologies revealed a decrease in PBMC IL‐10 production for egg allergic individuals,^107^ but an increase for those with milk allergy,^106^ both compared to NSNA controls. While these studies highlight differences in cytokine levels between specific allergies, they do not offer any insight into any concomitant behavioural or neurological effects that may be associated with IL‐10 alterations. Given that peripheral IL‐10 levels have been shown to be inversely correlated with general anxiety disorder scores,^114^ with its KO leading to anxiety‐ and depressive‐like behaviour in mice,^115^ future research may benefit from exploring such a potential relationship in the context of FA.

IL‐1β is commonly indicated as being upregulated, whether looking at FA in general (i.e., non‐specific allergen)^100^, ^101^, ^109^ or specifically at egg^103^ and peanut^108^ allergies. Of note, IL‐1β has been shown to directly affect enteric neurons,^116^ as well as negatively regulate human hippocampal neurogenesis.^117^

Elevated levels of IL‐6 have also been shown to negatively regulate hippocampal neurogenesis,^118^, ^119^, ^120^ and appear to be associated with the experience and risk of mental health disorders.^121^, ^122^, ^123^ In suspected and/or diagnosed FA patients, IL‐6 is primarily reported as being increased, based on PBMC expression following stimulation with LPS (specifically for egg allergy),^103^ or allergen (peanut allergy).^108^ mRNA from unstimulated PBMCs revealed a 100‐fold increase in IL‐6 expression for individuals who experienced anaphylaxis during a DBPCFC, but only for samples that were collected 1 week after the challenge.^100^ Contrarily, plasma samples collected 1 hr. after an OFC were shown to have decreased levels of IL‐6 for single‐egg or ‐peanut allergic patients compared to NSNA controls,^97^ perhaps indicating an effect of time after reaction.

Finally, TNF‐α levels have also been reported to be altered in FA, again in a manner related to specific allergens. While one study described increased expression of TNF‐α by peanut‐allergic, peanut‐stimulated PBMCs,^108^ another study described reduced levels of TNF‐α from egg‐allergic, OVA‐stimulated PBMCs,^107^ both compared to non‐allergic controls. Further complications arise when considering persistent and transient allergies, with Neeland et al.^103^ finding that TNF‐α levels were only significantly increased for the persistent egg allergy group, compared with both non‐allergic controls and also transient egg allergics. With rodent models indicating an increase in TNF‐α in the brain of FA individuals,^52^ uncovering the exact mechanisms associated with differential FA phenotypes will help to further understand their impact in a case‐by‐case manner.

### 3.2 Immune Cell Ratios

Beyond cytokine alterations, circulating immune cell ratios appear to be altered in FA. While the research on this limited, as most studies focus on PBMC cytokine production rather than cell proportions themselves, these changes present a potential avenue for future research into neuro‐immune interactions in FA—particularly given that research focusing on the association between peripheral immune cells and the CNS has rapidly gained traction over the past few years.

One particular population that appears to be altered in FA are CD4^+^ T cells, with changes observed from birth. Specifically, one study described an increase in the proportion of cord‐blood derived CD14^+^ monocytes over CD4^+^ T cells in children who received a FA diagnosis by 1 year of age, driven by a reduction in naïve Tregs compartments.^101^ Another study identified reduced percentages of CD4^+^CD25^hi^CD127^lo^Foxp3^+^ Tregs in food allergic children aged 0–6 years, though the effect was lost for children above this age. The authors emphasise the role of Tregs in the maintenance of allergen tolerance, a conclusion that is supported by findings from Klueber et al.^105^ In this study, peanut‐sensitised children with a negative OFC had increased Treg frequencies post‐OFC compared with baseline, whereas no such effect was described for OFC‐positive children. Others studies have demonstrated an increase in CD4^+^CD25^+^ Tregs in children with acquired tolerance to cow's milk^124^ and egg or peanut^125^ allergies. Interestingly, rodent models indicate that Treg migration to, and consequent proliferation in, the gut are required for the development of tolerance.^126^

Controversy persists regarding Treg populations in FA, exemplified by conflicting results from two studies by the same team focusing on the HealthNuts^127^ and SchoolNuts^128^ cohorts. Specifically, the former identified no differences in Treg populations among SA and non‐allergic groups, despite its young sample size (11 to 20 months). Conversely, the latter reported an increase in activated Treg frequency in single peanut allergic 10–14 year‐olds, compared to non‐allergics of a similar age. Thus, further research is required to elucidate the effects of specific FA, and single vs. multiple allergies on T cell populations of FA patients, as well as the association with age. Understanding these nuances will be crucial for understanding the downstream effects of such biological changes.

Proportions of CD14+ monocytes, being inversely correlated with that of CD4^+^ Tregs, appear to be increased in FA individuals.^101^, ^129^ Such changes to monocyte population proportions may be related to specific FA and transient vs. persistence allergy. While transient egg‐allergic infants showed increased percentages of circulating classical monocytes (HLADR^+^CD14^+^CD16^−^) compared with non‐allergic controls, those with persistent allergy also had increased proportions of nonclassical monocytes (HLADR^+^CD14^low^CD16^+^) and intermediate monocytes (HLADR^+^CD14^+^ CD16^+^), as well as myeloid DCs (HLADR^+^CD14^−^CD16^−^CD11c^+^CD123^−^).^103^ Higher proportions of classical DCs were also observed in both single‐peanut and multi‐food allergic participants compared to non‐FA controls.^128^ From the heterogeneous nature of FA, it is clear that further research is needed to truly define its mechanisms to allow the identification of immune patterns and profiles associated with the disorder.

While not explicitly explored within the context of FA, there is some evidence that alterations to circulating immune cell populations affects the brain and behaviour. In particular, CD4^+^CD25^+^ ^130^ and Foxp3^+^ ^131^ Treg‐depleted mice demonstrated increased anxiety‐like behaviours in the EPM and light–dark tests (respectively), and greater immobility (i.e., despair behaviour) during the forced swim test, as compared to control mice. Interestingly, Kim et al.^130^ further reported reduced serotonin levels in the hippocampus, under both stressed and normal conditions. Whether these effects exist in a FA‐specific context remains subject to future research.

## 4 UTILITY AND CHALLENGES OF UNDERSTANDING IMMUNE MECHANISMS OF FOOD ALLERGY

While the benefits of better defining FA mechanisms include alleviating patient burden and laying the groundwork for research on the wider effects of FA, there are still many barriers to doing so that mainly stem from the difficulty of exploring these mechanisms in vivo in humans.

Understanding the immune mechanisms of FA would directly benefit patients, through both aiding diagnoses and easing the burden of allergy management. The low to moderate specificity and positive predictive value of sIgE levels and the burdensome nature of OFCs denote that new diagnostic tools are needed. Of particular note is the basophil activation test (BAT), an in vitro assay that uses patient basophils to quantify the expression of CD63 (a marker of basophil activation^132^) after exposure to the allergen of interest.^133^ Unlike sIgE and SPT, BAT distinguishes between sensitised tolerance and allergy, as well as severity,^134^, ^135^, ^136^ potentially improving the scope of information that can be provided to patients and enabling tailored allergy management plans. Recently included in the EAACI guidelines on the diagnosis of IgE‐mediated FA,^13^ its high reported accuracy, specificity and sensitivity indicate that BAT has the potential to reduce the need of OFCs, thus further relieving patient burden.

Similarly, identifying immune profiles associated with FA onset would be useful to use in conjunction with, or even instead of, current diagnostic methods. For instance, one study found differences in cord blood mononuclear cells in children who received a FA diagnosis by 1 year of age, compared to those who did not,^101^ showing that such profiles may predate FA onset. Utilising this approach in high‐risk infants, for example, could help minimise the risk of accidental allergen exposure. Similarly, as explored in Section 4, novel bioinformatic approaches could enhance current diagnostic methods by assessing a patient's mediator profile, to possibly identify their specific allergy/ies,^97^, ^106^, ^107^, ^128^ the likelihood of allergy transience or persistence,^103^ as well as their risk of anaphylaxis.^100^ While such approaches require further research validation and methodological refinements to achieve feasibility of use in a healthcare setting, their value to the patient should not be understated, especially in light of the psychological impacts often described by FA patients and their caregivers, as previously outlined.^85^, ^86^, ^137^

Secondly, a deeper understanding of the mechanisms behind FA onset and persistence would facilitate targeted investigations into its broader effects, particularly regarding neuro‐immune changes. Disparities across the literature investigating such alterations in FA, such as cytokine levels and immune cell ratios and activation, render it difficult to fully decipher the scope and impact of interactions with the nervous systems. Nonetheless, such interactions do occur and not in isolation. For example, rodent models have demonstrated the emergence of anxiety‐like behaviours following FA induction, suggesting a direct effect of FA beyond the typical disease phenotype. With mental health changes already considered a burdensome aspect and an unmet need of FA by patients and caregivers,^138^, ^139^ it is clear that clarifying the biological processes behind these changes would enable future preventative strategies or treatments.

However, many barriers still exist to fully elucidating the immune mechanisms associated with FA (Box 2). Firstly, most studies that investigate FA mechanisms generally utilise rodent models, often wild‐type mice or rats—which do not spontaneously acquire FA.^140^, ^141^ Instead, sensitisation is induced, often in concurrence with an adjuvant, thus providing little utility on sensitisation itself.^142^ The extent of, and phenotype associated with, sensitisation can also differ based on the strain used, as shown by Mirotti et al.^71^ with their observed differences in sIgE between BALB/c and C57BL/6 mice following sensitisation and challenge to OVA. C57BL/6 mice also displayed a higher preference for sweetness, which may act as a confounding variable in studies exploring allergen avoidance. Other considerations involve sensitisation and challenge dosage, as well as route (intraperitoneal, nasal, oral, etc.), as reviewed by Bøgh et al.^143^ Thus, human ex vivo models, such as precision cut intestinal slice models which integrate immune cells and innervation in the GI‐tract,^144^ are essential to help translate some findings from rodent models into the human situation.

BOX 2 **Current barriers in food allergy research and avenues for future research**

**Table:** 

Current issues and barriers	Avenues for future research
Rodent models often require induced sensitisation, thus provide little utility on the process of attaining food allergies	Focus on spontaneous‐sensitised rodent models Further investigate the effects of mode of induced sensitisation (i.e. oral, nasal or percutaneous)
Allergic phenotypes in rodents can vary by sex and strain, potentially acting as a limiting factor for reproducibility across the literature	Ensure robust sample sizes are attained that include male and female animals Employ ex vivo human models to better translate findings from rodent models to the humans
Many studies do not capture transient vs. persistent allergy, despite these having been shown to have altered inflammatory profiles	Consider longitudinal designs with detailed allergy reports to better observe effects of allergy persistence
Food allergy does not present as a homogenous phenotype, with symptomatic differences arising across patients with single or multiple allergies, and even within patients	Include stringent allergy classification methods that capture the full atopic history of patients, not just focusing on the allergy of interest, to ensure a well‐defined allergic population Where possible, aim for sufficient samples sizes to allow examination across allergy phenotypes
The mechanisms driving increased anxiety levels in food allergy patients are little understood and often overlooked	Explore the biological mechanisms driving the emergence of anxiety in food allergy using currently available animal models Continue to develop and utilise food allergy‐specific anxiety scales, along with general and quality of life scales, in human studies to provide a better understanding of anxiety prevalence
Despite strong evidence of neuro‐immune interactions in the context of food allergy, there is still a gap in our understanding of their overall mechanisms and long‐term implications	Further establish tissue‐specific and systemic mechanistic models of neuro‐immune interactions in food allergy Expand focus of current rodent and human studies to include relevant psychological, cognitive, and neurological measures to decipher the effects of such neuro‐immune interactions
Studies into neuro‐immune interactions often focus on allergic reactions, overlooking the effects of more muted, but longer‐term, inflammatory changes associated with food allergy	Include sensitised controls without challenge as an experimental group when utilising sensitised/allergic mice Human studies include both baseline and allergic reaction‐associated assessment of cytokine levels—from circulating levels or PBMC production—and investigate the effects on in vitro neuronal cultures

Methodological variations do also exist across human ex vivo studies, however. Often, these involve PBMC isolation and stimulation to identify key cell types, their activation and expression. It's important to point out that studies differ on their use of stimulant; while many may use allergen, some also use LPS, to which reactions can vary based on environmental exposure.^108^, ^145^, ^146^ Similar to rodent models, human PBMC stimulation models may differ in dose and length of stimulation, and it is thus important to maintain this in mind when interpreting results.

Yet perhaps the largest challenge of studying FA mechanisms comes from disease's complexity. Patients experience diverse phenotypes and symptoms of differing severity, both across and within specific allergies. For instance, while tree nut and peanut allergies are generally considered persistent, cow's milk and hen's egg allergies can often be transient, with many children outgrowing their symptoms.^147^ Similarly, the symptoms experienced during a food allergic reaction are highly heterogeneous across patients—and even within patients with multiple FA—and severity of reactions vary without necessarily correlating with sIgE levels. Moreover, differences in cell ratios^128^ and plasma cytokine levels^97^ have been observed between single and multiple food allergic children, an extremely pertinent finding considering 40% of FA children are reported to have multiple FA.^148^ Thus, FA does not present as a single, homogenous phenotype and, resultantly, there is great need for future research to take these factors into consideration to best reflect the patient population. Other potential confounding factors that may affect the mechanisms associated with FA, such as age on the proportion of Tregs,^149^ or levels of platelet‐activating factor on risk of anaphylaxis,^150^, ^151^ should also be considered. Altogether, these challenges emphasise the requirement for comprehensive research with well‐defined allergic populations and sufficient sample sizes to best capture the heterogeneity of the disease.

## 5 CONCLUSION

FA diagnoses have been increasing in recent decades,^4^ yet our understanding of the disease mechanisms involved in its onset and persistence are not complete. In addition to the direct disease phenotype, it is becoming more and more apparent that FA may also lead to wider, downstream effects stemming from the underlying immune mechanisms. Of particular note, MCs and neurons bidirectionally communicate, with potential impacts on food allergic reaction symptoms and severity.^23^, ^45^, ^47^ FA‐associated changes to behaviour have also been reported, with allergen‐sensitised rodents demonstrating aversive^68^, ^69^ and anxiety‐like behaviours,^25^, ^89^, ^90^ and a timothy grass pollen sensitisation rodent model indicate potential alterations in AHN.^58^ However, the exact processes underlying these alterations are poorly understood, though some attention has been given to MC‐derived mediators^49^ and the cytokines associated with type‐2 immune response.^52^, ^58^ Still, with the heterogeneity associated with FA, there is a clear need for greater research to fully elucidate not only the wider systemic effects of the disease, but also the underlying mechanisms driving such changes, which can potentially be target therapeutically in the future.

## CONFLICT OF INTEREST STATEMENT

VH, EBF and ST report no conflicts of interest in relation to this manuscript. TE reports personal fees from Danone/Nutricia/Milupa, ThermoFisher, MADX, ALK, Novartis, Sanofi, EFSA outside the submitted work; non‐financial unconditional in kind support from MADX, Novartis and ALK, Co‐I or scientific lead in three investigator initiated oral immunotherapy trials supported by the Food Allergy and Anaphylaxis Program Sickkids and serves as associate editor for Allergy. Site PI of company sponsored trials by DBV, Novartis and Stallergen. RCK has received research funding from the National Institute for Health Research, Aimmune, National Peanut Board, Novartis and the Food Standards Agency and honoraria from Nutricia, Viatris and DBV Technologies. RCK is also Chair of the British Society for Allergy and Clinical Immunology Psychology Special Interest Group for Psychology. AFS reports grants from Medical Research Council (MR/M008517/1; MC/PC/18052; MR/T032081/1), Food Allergy Research and Education (FARE), the Immune Tolerance Network/National Institute of Allergy and Infectious Diseases (NIAID, NIH), Asthma UK (AUK‐BC‐2015‐01), BBSRC, Rosetrees Trust and the NIHR through the Biomedical Research Centre (BRC) award to Guy's and St Thomas' NHS Foundation Trust, during the conduct of the study; personal fees from Thermo Scientific, Nestle, Novartis, Allergy Therapeutics, IgGenix, Buhlmann, as well as research support from IgGenix, Buhlmann and Thermo Fisher Scientific through a collaboration agreement with King's College London.

## Data Availability

Data sharing is not applicable to this article as no new data were created or analyzed in this study.
